# Cleft Palate; Treatment of Simple Fractures by Operation; Diseases of Joints; Antrectomy; Hernia, Etc., Etc.

**Published:** 1899-05

**Authors:** 


CLEFT PALATE; Treatment of Simple Fractures by Operation; Diseases of Joints; Antrectomy; Hernia, etc., etc. A series of Clinical Lectures. By W. ARBUTHNOT LANE, M. S. Duodecimo, pp. 278. From the author. The Medical Publishing Company – Limited (Clinical Journal Office). 1898.

Here is a volume, small, neat, handy, which a busy man may pick up at odd times and find interesting. It is a literary nosegay, made up of fragrant blooms gathered in the garden of surgery. Mr. Lane's way of bunching up a handful of subjects is delightfully, refreshingly informal. As a matter of fact, this little volume has no name. Its title page contains simply this : "Cleft palate, treatment of simple fractures by operation, diseases of joints, antrectomy, hernia, etc., etc.," these and a few other surgical matters being lectures delivered by Mr. Lane at various times. While we may not agree with his views in every case, we must admire his delightful manner of expressing himself and the genuine ring of confidence which marks all his lectures. Cleft palate is spoken of at length and very cleverly handled and is profusely illustrated. Surgical procedure is carefully detailed in all his lectures and whenever possible is illustrated with drawings.

The lecture on acquired deformities is extremely interesting and includes "knock-knee, flat-foot, lateral curvature and dorsal excurvation"

Valuable and of interest are the lectures on mechanical or traumatic arthritis and tubercular affections of the joints. The lecture on treatment of simple fractures by operation is interesting, because it details the methods of operative procedure. Mr. Lane lectures this time secure in the belief that almost every simple fracture of long bones called for operative interference. While screwing, nailing or wiring together of fractures is required in some cases, American surgeons do not yet consider it necessary in every case. N. W. W.

